# Supplementary material for: Neuronal cell fate diversification controlled by sub-temporal action of Kruppel
Source: eLife. 2016 Oct 14;5:e19311. doi: 10.7554/eLife.19311 (PMC5065313; doi:10.7554/eLife.19311)
Supplement: Supplementary file 1. — DNA sequences for new UAS constructs. DOI: http://dx.doi.org/10.7554/eLife.19311.012 [file elife-19311-supp1.docx]

**Supplemental file 1: DNA sequences**

**Myc-Lbe**

gaattcaaccaaa**ATG**GAGCAGAAGCTGATCTCCGAGGAGGACCTGAACGAGATCCTGGGCGCCCCCTCCGGaGGtGGaGCCACCGCCGGaGCCGGtGGaGCCGGCGGCCCCGCCGGCCTGATCATGCTGTGCCCGCCAACAATGCGCCCCGCCAGCCCCGCCGAGAGCGAGATTAGCGTGGGAGGAGCCCCGAGCCCCCTGCCGACCCAGCATCACACCCATCCACACTCGCATCCACATCCCCTGCAACATCCCCGCGCCAGCACCATCGACCTGCTGCAACAGCAGCAGCTGCTGATGCAGCATCACGCCGCGGCAGCAGCTGCTGCCGCCGCTGCGGCAAGCGGCCTGACACGCTCGGCCGTGGGCAATCTGCCGGAGGATTATTTCCACCCCCTGAAGCGCCTGCGCATGTCGAGCAGTAGTAGCGAGCCACGCGACCATACCCCCTCGCCCCCATCCGCCGTGCCGGAGCCACAGACCAACCAGACAACCAAGTCGGCCATTGAGGGCGTGAAGAGCTTTTCCATCGCCGATATTCTGGGACACAGCGAGAAGCAGCGCGAGGAGAGTGTGAGCCCGCCCCCAAATGCCAACCTGCTGGCCCCCCCAGCCAGCCGCCCAATCGCCCCCTCGGGAGGCCTGCTGCAACCCCGCACCGAGCCCCTGGACGTGCATCCAGCCGCTGCCGCAGCCATGCTGCTGCCCAGCGGCCAGATTGTGCGCCCCTGGGATCACCTGCTGGGACCCACCATGCCAGTGCGCCCCTTCATCCCAAGCGCCCTGCTGCACTACGAGCAGCGCCTGGCCCTGGATTACCACCGCCAGCTGCAAGAGCACTTCAACGCCCAGGCCCAGCTGCTGCGCCACATGGGCATGAATCCGGCCATCATTGCCAGCGAGGATGGCAGCAGTGAGCGCTCGCAGCGCAGTTCGAGCAGTAACGGAAGCACCGAGTGCTGCTCGCCCCGCCAGGCCGAGAAGCTGGAGAAGCTGACAACCCAGGAGGGCTCGGAGGAGGCCCAGAAGAAGAAGTCCGAGGAGCAGCCCACCGGAAGCGGCAAGTCGAACGGAGATACGCCGCTGGATGCCCTGTTCCAGATGACAACCAAGGACTTCGACGAGTCGCAGGATAAGTCGCACCTGGACATCTTCAGCAACCGCCCCCAGCCGAAGAAGAAGCGCAAGAGCCGCACCGCCTTCACCAATCACCAGATTTTCGAGCTGGAGAAGCGCTTCCTGTACCAGAAGTACCTGAGCCCCGCCGACCGCGATGAGATCGCCGCCTCGCTGGGCCTGAGCAATGCCCAGGTAATCACCTGGTTCCAGAACCGCCGCGCCAAGCAGAAGCGCGATATCGAGGAGCTGAAGAAGGATTTCGACAGCGTGAAGGTGTTCTCCGCCCATAAGAGCTTTCTGGAGAATGTGAACGATCTGTCCATCCTGAAGAAGAAGCCCATGCACGAGTCCGATATGGTGGGACTGGCAGCCGCTGCGGCAGCAGCGGGAATGGTGGTGCCAGTGCCAGGAAGCGTGCCAATGGGAGGAGCCCCGCCAAAG**taatgatag**tctaga

**HA-Col**

gaattcaaccaaa**ATG**GCCGAGTGGGGCCGCAAGCTGTACCCCTCCGCCGTGTCCGGCCCCCGCTCCGCCGGCGGCCTGATGTTCGGCCTGCCCCCCACCGCCGCCGTGGACATGAACCAGCCCCGCGGCCCCATGACCTCCCTGAAGGAGGAGCCCCTGGGCTCCCGCTGGGCCATGCAGCCCGTGGTGGACCAGTCCAACCTGGGCATCGGCCGCGCCCACTTCGAGAAGCAGCCCCCCTCCAACCTGCGCAAGTCCAACTTCTTCCACTTCGTGATCGCCCTGTACGACCGCGCCGGCCAGCCCATCGAGATCGAGCGCACCGCCTTCATCGGCTTCATCGAGAAGGACTCCGAGTCCGACGCCACCAAGACCAACAACGGCATCCAGTACCGCCTGCAGCTGCTGTACGCCAACGGCGCCCGCCAGGAGCAGGACATCTTCGTGCGCCTGATCGACTCCGTGACCAAGCAGGCCATCATCTACGAGGGCCAGGACAAGAACCCCGAGATGTGCCGCGTGCTGCTGACCCACGAGGTGATGTGCTCCCGCTGCTGCGACAAGAAGTCCTGCGGCAACCGCAACGAGACCCCCTCCGACCCCGTGATCATCGACCGCTTCTTCCTGAAGTTCTTCCTGAAGTGCAACCAGAACTGCCTGAAGAACGCCGGCAACCCCCGCGACATGCGCCGCTTCCAGGTGGTGATCTCCACCCAGGTGGCCGTGGACGGCCCCCTGCTGGCCATCTCCGACAACATGTTCGTGCACAACAACTCCAAGCACGGCCGCCGCGCCAAGCGCCTGGACACCACCGAGGGCACCGGCAACACCTCCCTGTCCATCTCCGGCCACCCCCTGGCCCCCGACTCCACCTACGACGGCCTGTACCCCCCCCTGCCCGTGGCCACCCCCTGCATCAAGGCCATCTCCCCCTCCGAGGGCTGGACCACCGGCGGCGCCACCGTGATCATCGTGGGCGACAACTTCTTCGACGGCCTGCAGGTGGTGTTCGGCACCATGCTGGTGTGGTCCGAGCTGATCACCTCCCACGCCATCCGCGTGCAGACCCCCCCCCGCCACATCCCCGGCGTGGTGGAGGTGACCCTGTCCTACAAGTCCAAGCAGTTCTGCAAGGGCTCCCCCGGCCGCTTCGTGTACGTGTCCGCCCTGAACGAGCCCACCATCGACTACGGCTTCCAGCGCCTGCAGAAGCTGATCCCCCGCCACCCCGGCGACCCCGAGAAGCTGCAGAAGGAGATCATCCTGAAGCGCGCCGCCGACCTGGTGGAGGCCCTGTACTCCATGCCCCGCTCCCCCGGCGGCTCCACCGGCTTCAACTCCTACGCCGGCCAGCTGGCCGTGTCCGTGCAGGACGGCTCCGGCCAGTGGACCGAGGACGACTACCAGCGCGCCCAGTCCTCCTCCGTGTCCCCCCGCGGCGGCTACTGCTCCTCCGCCTCCACCCCCCACTCCTCCGGCGGCTCCTACGGCGCCACCGCCGCCTCCGCCGCCGTGGCCGCCACCGCCAACGGCTACGCCCCCGCCCCCAACATGGGCACCCTGTCCTCCTCCCCCGGCTCCGTGTTCAACTCCACCTCCATGTCCGCCGTGTCCTCCACCTGGCACCAGGCCTTCGTGCAGCACCACCACGCCGCCACCGCCCACCCCCACCACCACTACCCCCACCCCCACCAGCCCTGGCACAACCCCGCCGTGTCCGCCGCCACCGCCGCCGCCGTGGGCCCCCCCGGCTACCCCTACGACGTGCCCGACTACGCC**taatagtga**tttctaga
